# Significance of Targeting VEGFR-2 and Cyclin D1 in Luminal-A Breast Cancer

**DOI:** 10.3390/molecules25204606

**Published:** 2020-10-10

**Authors:** Ashraf N. Abdalla, Amal Qattan, Waleed H. Malki, Imran Shahid, Mohammad Akbar Hossain, Muhammad Ahmed

**Affiliations:** 1 Department of Pharmacology and Toxicology, College of Pharmacy, Umm Al-Qura University, Makkah 21955, Saudi Arabia; whmalki@uqu.edu.sa (W.H.M.); iyshahid@uqu.edu.sa (I.S.); mahossain@uqu.edu.sa (M.A.H.); mmsiddiqui@uqu.edu.sa (M.A.); 2 Medicinal and Aromatic Plants Research Institute, National Center for Research, Khartoum 2424, Sudan; 3 Breast Cancer Research, Department of Molecular Oncology, King Faisal Specialist Hospital and Research centre, Riyadh 11211, Saudi Arabia; akattan@kfshrc.edu.sa; 4 Department of Biochemistry and Molecular Medicine, School of Medicine and Health Sciences (SMHS), George Washington University, Washington, DC 20073, USA; 5 Department of Pharmacology and Toxicology, Faculty of Medicine, Umm Al-Qura University, Makkah 21955, Saudi Arabia

**Keywords:** luminal-A breast cancer, kinase activity profiling, VEGFR-2, Hsp90, CDK2, cyclin D1

## Abstract

The hormonal luminal-A is the most pre-dominant sub type of breast cancer (BC), and it is associated with a high level of cyclin D1 in Saudi patients. Tamoxifen is the golden therapy for hormonal BC, but resistance of cancer cells to tamoxifen contributes to the recurrence of BC due to many reasons, including high levels of AIB1 and cyclin D1. Overcoming drug resistance could be achieved by exploring alternative targetable therapeutic pathways and new drugs or combinations. The objective of this study was to determine the differentially enriched pathways in 12 samples of Saudi women diagnosed with luminal-A using the PamChip peptide microarray-based kinase activity profiling, and to compare the activity of HAA_2020_ and dinaciclib with tamoxifen in singles and combinations in the MCF7 luminal-A cell line. Our results of network and pathway analysis of the 12 samples highlighted the importance of VEGFR and CDKs in promoting luminal-A breast cancer. The activation of VEGF signaling via VEGFR-2 leads to activation of PI3K/AKT kinases and an increase of cell survival, and leads to activation of Hsp90, which induces the phosphorylation of FAK1, resulting in cytoskeleton remodeling. PLC-gamma 1 is also activated, leading to FAK-2 and PKC activation. Notably, the G_1_/S cell cycle phases and phosphorylation processes contribute to the top seven tumorigenesis processes in the 12 samples. Further, the MTT combination of HAA_2020_ and dinaciclib showed the best combination index (CI), was more clonogenic against MCF7 cells compared to the other combinations, and it also showed the best selectivity index (SI) in normal MRC5 cells. Interestingly, HAA_2020_ and dinaciclib showed a synergistic apoptotic and G_1_ cell cycle effect in MCF7 cells, which was supported by their synergistic CDK2, cyclin D1, and PCNA inhibition activities. Additionally, the combination showed VEGFR-2 and Hsp90 inhibition activities in MCF7 cells. The results show the significance of targeting VEGFR-2 and cyclin D1 in Saudi luminal-A breast cancer patients, and the effect of combining HAA_2020_ and dinaciclib on those targets in the MCF7 model. It also warrants further preclinical and in vivo investigations for the combination of HAA_2020_ and dinaciclib as a possible future second-line treatment for luminal-A breast cancers.

## 1. Introduction

Breast cancer (BC) is a global health challenge. With 11.6% of the total diagnosed cancer cases in 2018 in 185 countries, it remains the number one malignancy in females [1]. In Saudi Arabia, it is also the top female malignancy in all ages, reaching 28.7% in 2014 [2], which is more than double the average global incidence percentage. BC comprises four sub-types, determined by the hormone receptor (HR) and human epidermal growth factor receptor 2 (Her2). The hormonal luminal-A sub-type (HR^+^/Her2) is the most dominant, which affected more than 73% of the total BC patients in the US alone during 2019–2020 [3]. In a study of 359 Saudi BC patients, luminal-A cases showed the highest percentage (58.5%). Similar characterization studies in different countries revealed variation of the luminal-A percentage. As an example, an average of 34.7% was reported in Oman, 69% in Poland, and 47.4% and 54% for African and non-African Americans, respectively, during the period of 1993–2010 [4]. In a Saudi BC molecular profiling study, luminal CK8/18 (normal luminal epithelial like differentiation 8/18) and some genes, mainly cyclin D1 and LIV1 (Zin transporter LIV1), were reported to be associated with the luminal-A sub-type [5].

Up to date, the most important available treatment options for luminal-A are hormonal therapies either alone or in combinations, including tamoxifen, fulvestrant, exemestane, and aromatase inhibitors (AIs). In addition to the mammalian target of rapamycin (mTOR) inhibitor everolimus, and some chemotherapeutic agents, including the microtubule inhibitors (taxanes and vinca alkaloids), anthracyclines, gemcitabine, capecitabine, and cyclophosphamide. The combination of paclitaxel and bevacizumab (anti-VEGFR-A inhibitor) is used in aggressive cases. Additionally, the CDK 4/6 inhibitor palbociclib can be considered for luminal-A cases [6]. As concerns tamoxifen, a member of the selective estrogen receptor modulators (SERMs), it is regarded as the golden drug for the luminal-A sub-type. It possesses a high therapeutic effect, which is shown by binding with the estrogen receptor (ER), causing an antiproliferative effect on mammary cells [7,8,9,10]. However, resistance to tamoxifen contributes to the recurrence of BC for more than 25% of its users, thus justifying the use of second-line drugs, such as selective estrogen receptor degraders (SERDs), or AIs [11]. Many cellular mechanisms may explain resistance to tamoxifen, which can either be intrinsic due to cytochrome p450 (Cyp450) nucleotide polymorphisms leading to disrupted tamoxifen metabolism or acquired after the commencement of tamoxifen therapy [11,12]. There are various pathways through which an acquired resistance is derived, mostly involving the loss of ER expression, which consequently halts tamoxifen effects. Additionally, the high expression level of co-activators, like SRC-3, AIB1, or TRAM-1, may result in the conversion of tamoxifen from an antagonist to agonist, again leading to deprivation of its therapeutic role in BC. Specifically, AIB1 overexpression is linked with higher levels of cyclin D1 [11]. Moreover, the phosphorylation of ER by Akt and MAPK leads to its activation. Overcoming anti-ER drug resistance may be achieved by the discovery of new therapeutic pathways, drug classes, and combinations that can help patients beyond palliation. Munzone et al. highlighted that the acquired tamoxifen resistance to ER may be reverted by its combination with EGFR inhibitors [11,13,14].

The use of high-throughput technologies, including tandem mass spectrometry, RNA-microarrays, and RNA sequencing, results in large data sets that are useful in the determination of differentially enriched pathways, upstream/downstream proteins, and regulators [15]. Consequently, such -omics studies may help in overcoming drug resistance by discovering new or important therapeutic pathways, thus realizing cancer-personalized therapies. In a transcriptomic study of six BC patients from Qatar, next-generation sequencing (NGS) was used as a tool for the identification of upregulated and downregulated transcripts. The differentially expressed transcripts through RNA-Seq and qRT-PCR were further investigated using ingenuity signaling pathway analysis (PA), revealing that the most important activated mechanistic networks were Her2, IGFBP2, ESR1, and FOXM1 [16]. Another study, which aimed at studying the transcriptomic profile of 45 Saudi BC samples of different types, resulted in 544 upregulated and 615 downregulated genes compared to normal breast tissues. The top detected gene groups in that study were cell division cycle-6 homolog (*S. cerevisiae*) (CDC6), chemokine (C-X-C motif) ligand-10 (CXCL10), and topoisomerase-2α (TOP2A) among others. Additionally, the PA highlighted the role of the cell cycle, ATM signaling, DNA damage, glycerolipid metabolism, and the ILK signaling pathways [17]. Adnan et al. also highlighted that the regulators of the cell cycle, including cyclin B1, cyclin B2, and CDK1, and estrogen-dependent BC signaling pathways (including MRAS, PIK3C2G, AKT3, CREB3L4, CREB5, and EGFR) are some of the most important canonical pathways predicted by ingenuity PA in Saudi BC samples [18].

One of the relatively new methods for understanding signaling pathways is PamChip peptide micro-array-based kinase activity profiling, which is used to study kinase activity in complex media, like cell lysates, without prior knowledge of the pathways involved. The PamChip workstation uses a flow-through micro-array system on which immobilized peptides can be phosphorylated by kinases found in, e.g., a clinical sample, in the presence of ATP [19,20,21]. The amino acid sequences of these peptides are derived from known phosphorylation sites of human proteins. With the two available assays, phosphorylation by tyrosine kinase (PTK) or serine/threonine kinase (STK) of 144 immobilized peptides can be measured. Peptide phosphorylation is then detected with either an FITC-labeled anti-phosphotyrosine antibody for PTK application or a mixture of anti-phosphoserine/threonine antibodies in combination with an FITC-labeled secondary antibody for STK application.

The objective of this study was to determine the most important targetable kinase pathways and networks that are differentially upregulated in collected Saudi hormonal luminal-A clinical samples using PamChip pathway analysis. In addition, based on this result, the multi-kinase inhibitor (compound 1 “HAA_2020_”: [22]) and the pan CDK inhibitor (dinaciclib) were used to target the resulting pathways in MCF7 cells as a model for luminal-A breast cancer. The effect of HAA_2020_ and dinaciclib was compared with tamoxifen in single and combined treatments.

## 2. Results

### 2.1. Characteristics of Clinical Samples and Ethical Clearance

The characteristics of the samples included in this study are shown in Table 1. Most of the clinical sample characteristics under study were similar to the characteristics of previous Saudi studies [4,5], as 50% of the samples were from pre-menopausal patients, and 66% and 34% were grade II and III, respectively. Additionally, 83% were ductal vs. 17% lobular samples. Most samples in this study showed over 60% ER, whereas more than 50% showed less than 19% PR and Ki-67.

### 2.2. Kinase Activity in Luminal-A Breast Tumors

Tyrosine and serine/threonine kinase activity were determined in this study using the PamChip peptide microarrays in Saudi luminal-A breast cancer samples for the first time to our knowledge. The biological variation between the samples was large. The results of the assays were combined, analyzed as described in the Section 1, Section 4, and uploaded into Metacore. The Metacore enrichment analysis consisted of matching highly phosphorylated protein identities in the 12 luminal-A samples with gene identities in the functional ontologies in MetaCore. The probability of a random intersection between the set of identities and the target list with the ontology entities is estimated as a *p*-value of hypergeometric intersection. A lower *p*-value means a higher relevance of the entity to the dataset, which is shown as a higher rating for the entity.

#### 2.2.1. Network Analysis

Tumorigenesis is a multi-faceted process, involving a complicated network of interactions and many kinases. The width of the network is illustrated by the network and PA. The Metacore network analysis indicated that VEGF signaling via the VEGFR-2 pathway is one of the top important networks involved in the tumorigenesis of 12 luminal-A samples, involving 56 different pathways. The ephrin receptors involved in cell–cell adhesion also ranked high, among other tyrosine kinases (Table 2).

**Figure 1:**
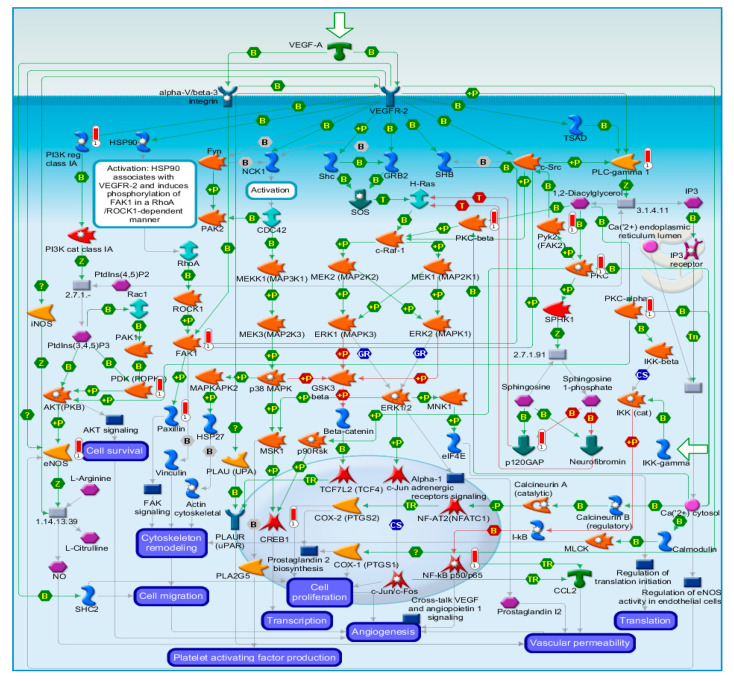
Pathway analysis map showing VEGF signaling via VEGFR-2 generic cascades. Experimental data are projected onto and visualized on the maps as thermometer-like figures. Upward thermometers are shown in the red color and indicate upregulated phosphorylation levels of the proteins (key of the map: Supplementary Material Figure S1).

The VEGFR-2 pathway map (Figure 1) shows that the activation of VEGFR-2 leads to activation of the PI3K/AKT signaling pathway, causing an increase of cell survival. Additionally, it leads to activation of heat shock protein 90 (Hsp90), which induces phosphorylation of FAK1 in a RhoA/ROCK 1-dependent manner all leading to cytoskeleton remodeling. PLC-gamma 1 is also activated, leading to FAK-2 and PKC activation. Similar to the ephrins, FAK is important for cell–cell adhesion.

#### 2.2.2. Process Network

**Figure 2:**
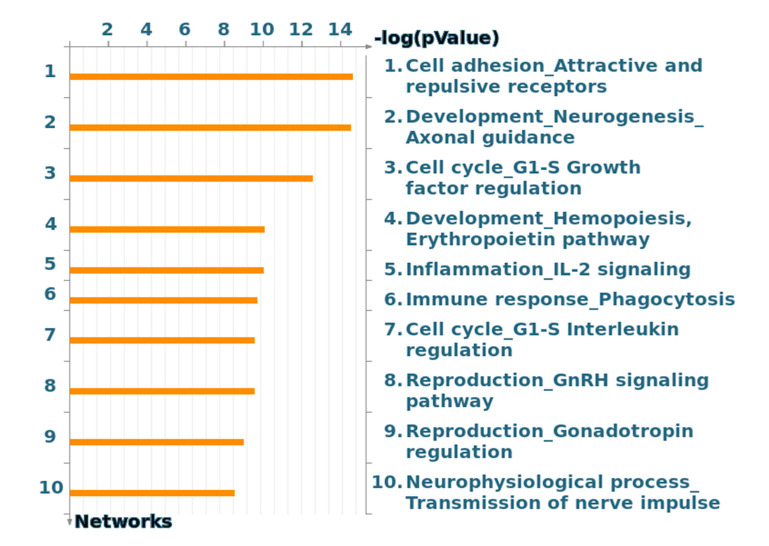
Top 10 process networks based on data obtained from the kinase activity measurements on PamChip.

The analysis of cellular and molecular processes of the 12 samples is presented in Figure 2. Each process represents a pre-set network of protein interactions characteristic for the process. Cell–cell adhesions scored high in this analysis. Noteworthy, the cell cycle G_1_/S growth phases contribute to two of the top seven processes.

#### 2.2.3. GO Processes

**Figure 3:**
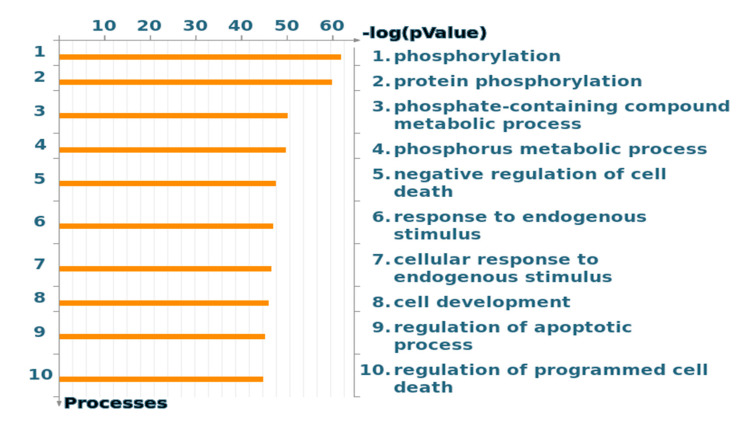
Gene ontology (GO) processes, sorted by statistically significant processes.

The gene ontology (GO) cellular processes of the 12 samples also showed the importance of the phosphorylation process (Figure 3), which was expected since kinase activity has been determined to generate the input for Metacore. Cell cycle CDKs are among the important kinases (Supplementary Material Table S2 and File S3).

In conclusion, based on the Metacore analysis, the processes involving VEGFR-2, Hsp90, G_1_/S, and CDKs are important in promoting luminal-A breast cancer in the 12 Saudi samples.

### 2.3. Combination Cytotoxicity and Selectivity Studies

Based on the previous PA results of this study, the multi-kinase inhibitor HAA_2020_ (previously described novel quinazoline that showed potent EGFR, VEGFR-2, Her2, and Hsp90 inhibition activities [22,23]) was selected for combination studies with tamoxifen using the MTT assay. Additionally, to address the importance of the cell cycle G_1_/S phases, the pan CDK and cyclin D1 inhibitor dinaciclib was selected for the comparative MTT combination studies with tamoxifen and HAA_2020._ First, the MTT assay was performed for each of the three compounds, and the IC_50_ values are shown in Table 3. The IC_50_ of HAA_2020_ was similar to a previous report in MCF7 cells: 480 nM [22]. Following this, a combination study was performed in MCF7 cells (72 h) between HAA_2020_ and tamoxifen (this ratio was selected based on the two compounds’ IC_50_ values: 1:1.5, respectively) which showed synergistic activity (CI: 0.964, calculated using Compusyn). Further, the combination between HAA_2020_ and dinaciclib (100:1) exerted better CI at the lowest concentration of each compound (CI: 0.463). Contrary, the combination of tamoxifen and dinaciclib (150:1) caused antagonistic activity at the lower concentrations, but at higher concentrations, it exerted weak synergistic activity. Finally, the combination of the three compounds also showed synergism. However, the best combination was between HAA_2020_ and dinaciclib (Table 3). On the next step, the selectivity of the three compounds and their combinations was tested using MRC5 cells, and the selectivity index (SI) was calculated. Dinaciclib showed the best selectivity towards MCF7 cells compared to MRC5 cells, followed by HAA_2020_ and tamoxifen (SI: 62, 32, and 25, respectively). Whereas, the combination of HAA_2020_ and dinaciclib showed the best SI, which agrees with its highest CI compared to other combinations, followed by the combination of the three compounds, the combination of HAA_2020_ and tamoxifen, and finally, tamoxifen and dinaciclib.

### 2.4. Clonogenicity of the Three Compounds and their Combinations

**Figure 4:**
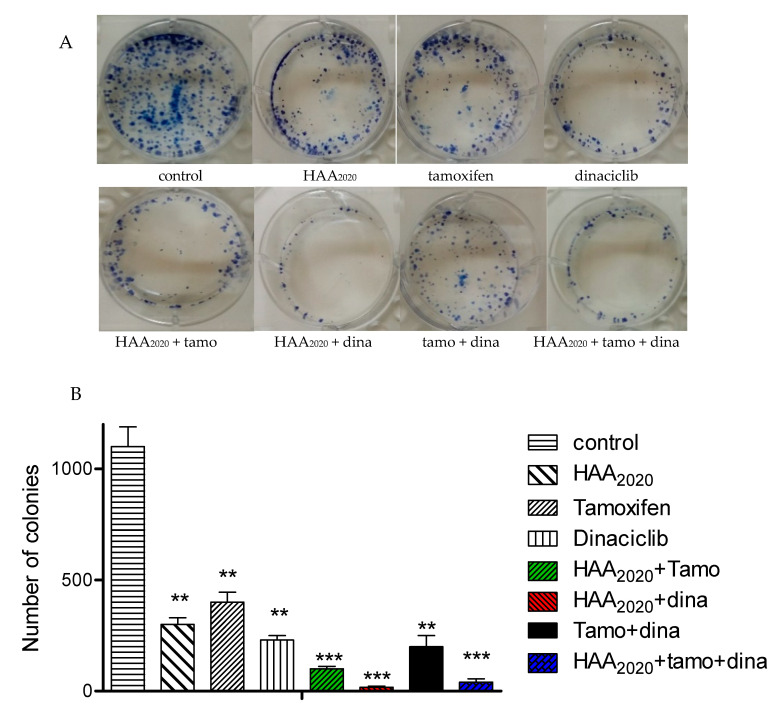
Clonogenicty of HAA_2020_, tamoxifen, dinaciclib, and their combinations in MCF7 cells (24 h), followed by 14 days of drug-free incubation. (**A**) MCF7 colonies in 6-well plates stained with methylene blue. (**B**) Bar figure showing the effect of each compound/combination (*n* = 3). Experiments were repeated ×3 times. Statistical differences compared to untreated control cells were assessed by one-way Anova with the Tukey’s post-hoc multiple comparison test. *p* < 0.01 (**) and *p* < 0.001 (***) were taken as significant.

The clonogenic survival assay was performed to confirm the growth inhibitory effect of HAA_2020_ (500 nM), tamoxifen (750 nM), dinaciclib (5 nM), and their combinations. Following the addition of the compounds, the colony number of MCF7 cells was significantly decreased by each of the three compounds, mainly by dinaciclib, followed by HAA_2020_ and tamoxifen. Furthermore, the combination of HAA_2020_ and dinaciclib showed the strongest clonogenic activity, followed by the combination of the three compounds (Figure 4).

### 2.5. Cell Cycle Analysis

**Figure 5:**
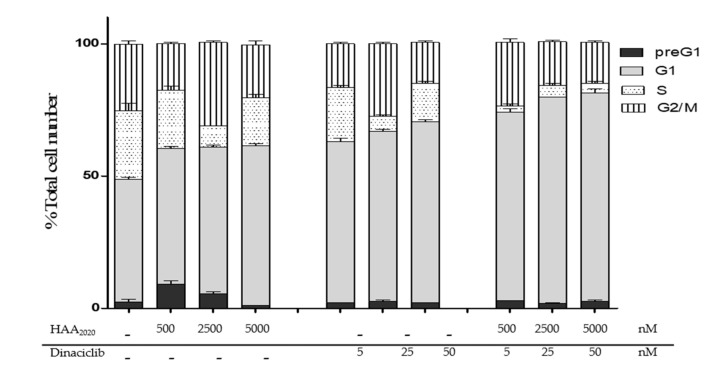
Cell cycle analysis in MCF7 cells treated for 24 h with either HAA_2020_ (500, 2500, 5000 nM), dinaciclib (5, 25, 50 nM) or their combinations. Cell cycle phases: preG_1_, G_1_, S, and G_2_/M. Data is represented as Mean ± SD (*n* = 3, two independent experiments). Histograms of 20,000 events are presented in supplementary material S4.

The combination of HAA_2020_ and dinaciclib showed the strongest CI and SI, and they were more clonogenic compared to the combination of HAA_2020,_ tamoxifen, and dinaciclib all together. Thus, HAA_2020_ (500, 2500, and 5000 nM) and dinaciclib (5, 25, and 50 nM) were selected for further investigations to explore the mechanisms that could justify their synergism. First, the cell cycle distribution assay was performed to study each HAA_2020_ and dinaciclib antiproliferative effect in MCF7 cells (24 h), which elicited a dose-dependent arrest of cells in the G_1_ phase, in addition to a slight increase of cells in the G_2_/M phase in their second concentration (Figure 5). Moreover, their combination showed a higher increase in the G_1_ phase, all at the expense of other cell cycle phases. This effect agrees with the importance of targeting G_1_/S phases, which was highlighted by the PamChip process analysis of the 12 BC samples.

### 2.6. Detection of Apoptosis

**Figure 6:**
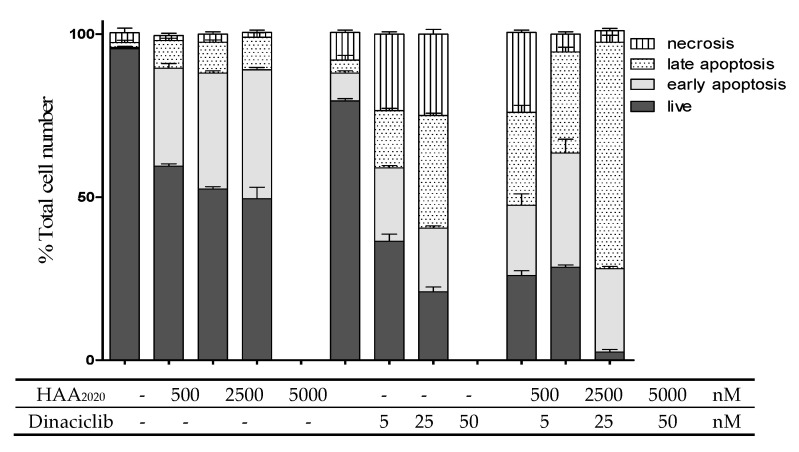
Detection of apoptosis in MCF7 cells (Total number %, 24 h). Cells were treated with either HAA_2020_ (500, 2500, 5000 nM), dinaciclib (5, 25, 50 nM) or their combinations. Cells were stained with annexin V FITC/PI. A total of 20,000 single-cell events were acquired on a BC-500 flow cytometer and analyzed by Expo 32 software. Data is represented as mean ±SD (n = 3, two independent experiments). Cell staining status: necrotic cells (annexin V−/PI+), late apoptotic cells (annexin V+/PI+), live cells (annexin V−/PI−), early apoptotic cells (annexin V+/PI−). Histograms are shown in the supplementary material figure S5.

HAA_2020_ and dinaciclib were further used to treat MCF7 cells (24 h) to investigate their apoptotic-inducing activity. HAA_2020_ (500, 2500, 5000 nM) caused a dose-dependent increase of early apoptosis compared to the control (0.5–40.0%), and also late apoptosis (2.0–10.0%). Dinaciclib also caused an increase of the early apoptotic event compared to the control, but it was half of that caused by HAA_2020_ (0.5–20.0%). Notably, dinaciclib caused more late apoptosis in MCF7 cells compared to the control (2.0–35.0%). The combination of HAA_2020_ and dinaciclib caused early apoptosis similar to the amount caused by dinaciclib alone (0.5–24.0%), while it caused double the late apoptotic event of dinaciclib alone (1.0–70.0%). Taking early and late apoptosis together, the combination of HAA_2020_ and dinaciclib caused the highest induction of apoptosis compared to each of the compounds alone. Thus, HAA_2020_ and dinaciclib and their combination were proved to induce significant apoptosis in MCF7 cells in a dose-dependent manner (Figure 6).

### 2.7. Western Blotting of CDK2 and Cyclin D 1 Proteins

**Figure 7:**
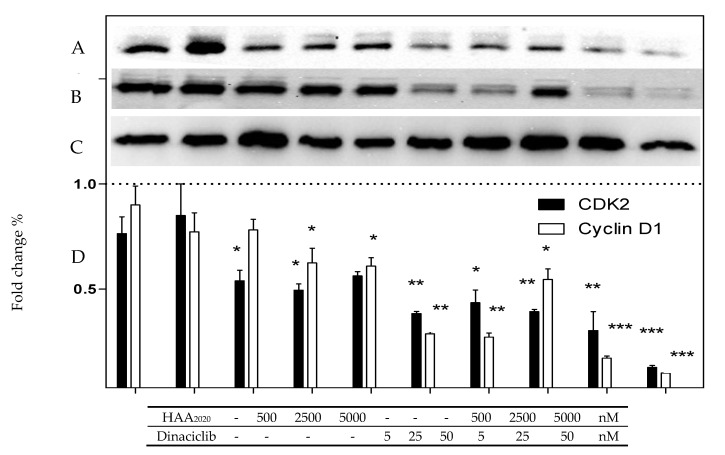
Level amount of CDK2 and Cyclin D1 in MCF7 cells treated with either HAA_2020_ (500, 2500, 5000 nM), dinaciclib (5, 25, 50 nM), or their combinations for 24 h. (**A**–**C**): immunoblots of CDK2, cyclin D1, and GAPDH, respectively. (**D**): Data is represented as mean ±SD (*n* = 2, two independent experiments). Statistical differences of the relative protein levels normalized to GAPDH were assessed by one-way Anova with the Tukey’s post-hoc multiple comparison test. *p* < 0.05 (*), *p* < 0.01 (**), and *p* < 0.001 (***) were taken as significant. Image J software was used for densitometry.

The effect of HAA_2020_ and dinaciclib (24 h) on the level of CDK2 and its partner cyclin D1 in MCF7 cells was investigated using Western blotting, in line with the effect of HAA_2020_ and dinaciclib on the G_1_/S cell cycle phase. HAA_2020_ caused a significant decrease of the CDK2 level compared to the control GAPDH at its highest dose (5000 nM) while dinaciclib caused a more significant dose-dependent decrease of CDK2 compared to HAA_2020._ The combination of HAA_2020_ and dinaciclib synergized effectively in decreasing CDK2 at all the used doses, especially the highest doses (Figure 7). Regarding cyclin D1, HAA_2020_ caused a non-significant decrease of the cyclin D1 level compared to the control GAPDH. Contrarily, dinaciclib again caused a more significant dose-dependent decrease of Cyclin D1 compared to HAA_2020._ However, the combination of the two drugs caused a significant decrease of cyclin D1, which is attributed to the effect of dinaciclib.

### 2.8. Real-Time PCR

**Figure 8:**
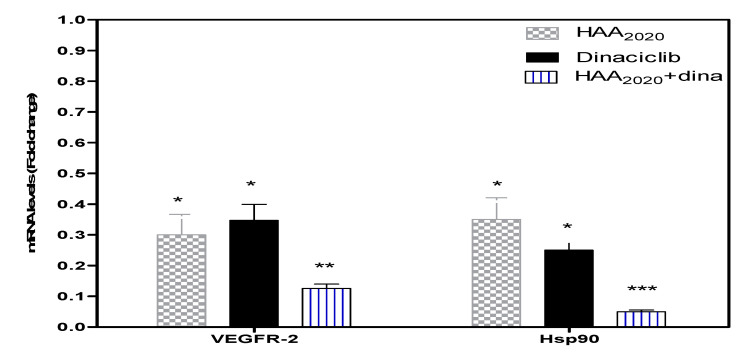
The expression of VEGFR-2 and Hsp90 genes in MCF7 cells. MCF7 cells were treated for 24 h with either HAA_2020_ (500 nM), dinaciclib (5 nM), or their combination. Data is represented as mean ±SD (*n* = 3, two independent experiments). The results are expressed as fold-change compared with the untreated group (1-fold change). The raw delta-Ct values (the difference between CT values obtained for the gene of interest and house keeping gene) were converted into relative expression levels (fold-change) using the formula 2^−∆∆Ct^. Statistical differences, compared to untreated control cells, were assessed by one-way Anova with the Tukey’s post-hoc multiple comparison test. *p* < 0.05 (*), *p* < 0.01 (**), and *p* < 0.001 (***) were taken as significant.

Real-time PCR was performed to investigate the mRNA amount of VEGFR-2 and Hsp90 genes in MCF7 cells following treatment with either HAA_2020_ (500 nM), dinaciclib (5 nM), or their combination for 24 h. HAA_2020_ and dinaciclib significantly downregulated VEGFR-2 and Hsp90, and the combination showed better inhibition compared to each of the compounds alone (Figure 8).

### 2.9. Immunofluorescent Imaging

**Figure 9:**
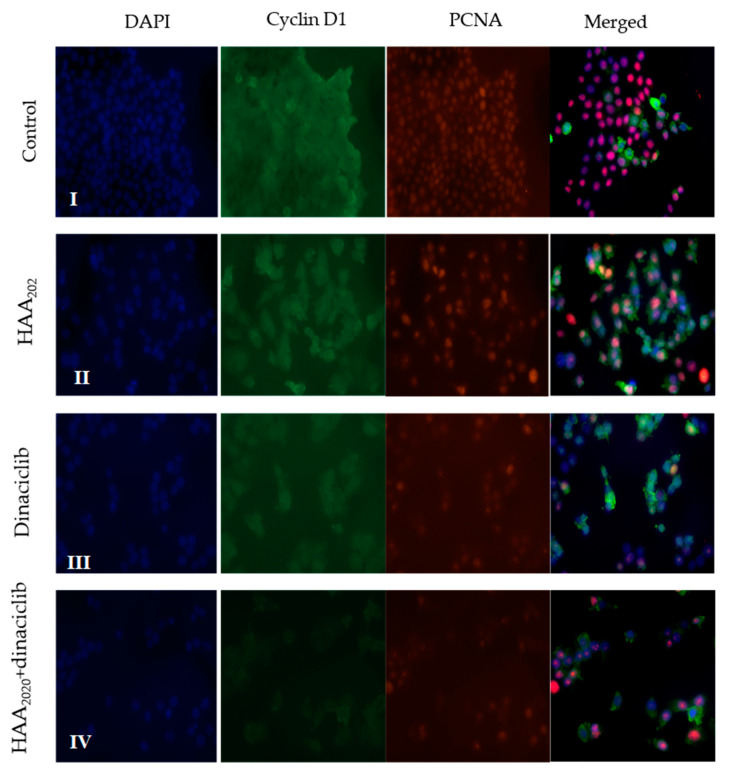
The co-localization of the immunofluorescent bodies using cyclin D1 (green) combined with PCNA (red), counterstained with DAPI (4‘,6-diamidino-2-phenylindole) in MCF7 cells (scale bar = 15 μm; 40× objective). **I**: untreated control, **II**: cells treated with HAA_2020_, **III**: cells treated with dinaciclib, **IV**: cells treated with both HAA_2020_, dinaciclib.

The immunofluorescence of MCF7 cells treated with HAA_2020_, dinaciclib, or their combination for 24 h was investigated. The control cell panel demonstrated the co-localization of cyclin D1 and the proliferating cell nuclear antigen (PCNA) cell cycle regulatory protein (Figure 9 I). Treatment of cells with HAA_2020_ (500 nM) decreased cyclin D1 (green) and PCNA (red, Figure 9 II) while treatment of cells with dinaciclib (5 nM) caused more inhibition of cyclin D1 and PCNA compared to the control and HAA_2020_ (Figure 9 III). In agreement with the previous results, the combination of HAA_2020_ and dinaciclib exhibited a more pronounced inhibition of the cyclin D1 and PCNA proteins (Figure 9 IV).

## 3. Discussion

The 12 clinical samples in this study were randomly collected from Saudi women diagnosed with hormonal luminal-A breast cancer to investigate the most important targetable kinase pathways, and to compare the effect of HAA_2020_ and dinaciclib to tamoxifen on those targets in MCF7 cells. The characteristics of the 12 samples were similar to previously reported Saudi luminal-A characteristics [4,5]. Notably, most of the samples in this study showed over 60% ER, and for this type of cancer, tamoxifen can be used as the first-line drug. However, the possible resistance to tamoxifen requires the exploration of further treatment options [11].

The investigation of the most targetable therapeutic pathways may help in finding more suitable known alternatives to tamoxifen, new drug(s), or new drug combinations. For this purpose, we used the PamChip peptide microarray-based kinase activity profiling assay to determine the most important kinase pathways in the whole-cell lysate of the 12 luminal-A samples, which resulted in highlighting the importance of the G_1_/S cell cycle phase, CDKs, and the VEGFR-2 pathway, leading to the activation of PI3K/ AKT and Hsp90 and induction of FAK phosphorylation. All these pathways end up with cell migration, cell proliferation, and angiogenesis.

Angiogenesis is one of the important hallmarks of cancer [24], and ER is an essential agonist and inducer of angiogenesis signaling pathways [25]. Angiogenesis can be inhibited by the VEGFR-2 signaling pathway inhibitors like sorafenib or sunitinib. Additionally, aflibercept showed anti-VEGFR-2 clinical efficiency when combined with cytotoxic agents mainly due to the effect on the endothelial cell cycle. Interestingly, the anti-VEGF-A drug bevacizumab failed to improve patients’ overall survival as a monotherapy at first, thus it was combined with paclitaxel for the HER2^-^ metastatic BC and got Food and Drug Administration (FDA) approval, before withdrawal of this combination again due to efficacy issues and acquired resistance. Thus, new combinations that can show stronger effects on major hallmarks of cancer, like the cell cycle and apoptosis, are needed to overcome the drawbacks of the previous anti-VEGFR combinations [24].

As concerns the relationship between tamoxifen resistance and Hsp90 overexpression, tamoxifen and its active metabolite 4-hydroxytamoxifen were shown in a study to be Hsp90 ligands, thus increasing its ATPase activity [26]. The overexpression of Hsp90 was also associated with the large size of BC tumors, and it is an important protein for the maintenance of EGFR, AKT, MET, and VEGFR [27]. Additionally, ER and the cell division control proteins: CDK1, CDK2, Cdc4, and Cdc6 are all among the Hsp90 protein clients [28,29]. Numerous tyrosine kinase inhibitors (TKIs) with Hsp90 inhibition activity exert their actions by inhibiting cancer cells in the G_1_/S and G_2_/M cell cycle phases, and upregulation of p53. Consequently, Hsp90i is an intriguing area for drug discovery and development, because of the multiple protein interactions leading to apoptosis or differentiation of the cancer cell [27,28,30]. In a review of more than 15 clinical trials (stage II), including resistant cases to standard anticancer drugs, Hsp90i showed promising results in different malignancies. Four of the trials were dedicated for BC patients investigating the therapeutic outcome of 17-AAG (alone or combined with trastuzumab or ganetespib), or IPI-504 (combined with trastuzumab). ER^+^/Her2^-^ or Her2^+^ were the targeted genotypes in the ganetespib BC clinical trial [31]. These clinical findings agree with the results of a retrospective study showing that the best clinical efficacy of Hsp90i was seen in metastatic BC [30]. In another study, geldanamycin was found to inhibit cyclin E/CDK2 and CDK4 in human prostate (DU145) and human colorectal (HT29) cancer cell lines [32], and it showed synergistic activity with the CDK 4/6 inhibitor (palbociclib) in HCT116 colon cancer cells [33].

Many quinazolines were designed as multiple TKIs, including vandetanib which targets VEGFR/EGFR, and cediranib, which targets VEGFR/PDGFR. Additionally, afatinib, gefitinib, erlotinib, and lapatinib were designed to target EGFR and/or Her2 [34]. Some other quinazoline derivatives were designed as Hsp90i like 3-phenyl-2-styryl-3H-quinazolin-4-one, which was approved against BC [35]. Alkahtani et al. synthetized novel quinazoline derivatives, from which compound 1 (4-(2-(4-Oxo-2-thioxo-1,4-dihydroquinazolin-3(2*H*)-yl)ethyl) benzene sulfonamide) showed multi-tyrosine kinase inhibition activity against EGFR, VEGFR-2, and Her2 kinases (0.43, 0.34, and 0.15 µM, respectively) compared to sorafinib against the same kinases (0.11, 0.17, and 0.13 µM, respectively). The molecular docking of compound 1 showed that the quinazoline ring was essential for its anti-kinase activity, and that the addition of the acetanilide moiety to benzenesulfonamid-containing quinazolinone was an important step towards the elevation of its enzyme activity. Compound 1 also showed cytotoxicity against MCF7, HL60, K562, and HT29 cancer cell lines, and displayed selectivity towards these cancer cells compared to MRC5 normal cells, all of which was comparable to sorafinib. The IC_50_ of compound 1 against MCF7 was better compared to sorafinib against the same cells (0.65 and 2.50 µM, respectively) [22]. In another study, the Hsp90 inhibition activity of compound 1 (named HAA_2020_) was tested using the surface plasmon resonance (SRB). The interaction between HAA_2020_ and Hsp90α scored 51 nM K_D_, thus showing affinity towards the chaperone, which was better than the affinity shown by the standard drug: 17-AAG [23,36]. For these reasons, HAA_2020_ was selected to be investigated in this study compared to tamoxifen.

The analysis of the cellular and molecular processes of the 12 luminal-A samples represented the protein interactions for the main processes leading to BC. It was also found that the cell cycle G_1_/S growth process contributes to two of the top seven processes. Additionally, the GO processes showed the importance of kinase phosphorylation to the tumorigenesis process of the luminal-A samples. Previously, some studies highlighted the importance of CDKs and cyclin D1 to Saudi BC patients [5,18]. Additionally, in another study that included 100 Saudi women with BC, cyclin D1 was found in 68% of the cases, which was significantly co-related with ER and PR status [37].

Targeting CDKs and their partner proteins (i.e., cyclin D1) previously showed promising results, because of their ability to control the cell cycle and cellular transcription. The ATP-competitive inhibitor, dinaciclib, exhibited IC_50_ sub 4 nM against a broad spectrum of CDKs (1, 2, 5, and 9) and RB phosphorylation in BC cell lines, simultaneously with induction of apoptosis, all of which was better compared to flavopiridol. It was also reported that the combination of dinaciclib with an anthracycline showed synergism in BC cell lines [38]. It also showed encouraging efficacy, safety, and tolerability profiles as a monotherapy in a clinical trial phase II for advanced BC, which involved ER^+^/Her2^-^ patients. Moreover, it was concluded in a clinical study that the investigation of more targeted combinations with dinaciclib for BC could overcome toxicity [39,40,41]. In another previous report, dinaciclib showed a synergetic relationship with ABT-737, a tyrosine kinase inhibitor that targets the Bcl-2 family [42]. For all these reasons, dinaciclib was also selected in this study in combination with HAA_2020_ and tamoxifen.

One of the most decisive factors to consider in a pathway or network analysis is the biological relevance to the disease. The result of PA in this study showed the importance of CDKs and the VEGFR-2/PI3K/AKT/Hsp90 pathways in addition to the importance of the cell cycle G_1_/S growth and phosphorylation processes. Consequently, HAA_2020_ and dinaciclib were selected for MTT combination studies with tamoxifen, which showed that the best CI was for the combination of HAA_2020_ and dinaciclib in MCF7 cells, followed by the combination of the three compounds. The main reason attributed to the failure of CDK inhibitors in the clinic is their toxicity in normal cells along with cancerous cells [43]. Thus, MRC5 normal cells were used in this study to investigate the selectivity of the three compounds and their combinations, and the results showed that the combination of HAA_2020_ and dinaciclib was the least toxic against MRC5 cells compared to the other combinations. However, while the MTT assay is rapid, precise, and non-radioactive, it can rarely give false-positive results, as the mitochondrial reductase enzymes may be inhibited by other factors, including superoxide generation [44]. Additionally, some drugs may interfere directly with MTT reduction [45]. Thus, the clonogenic assay was performed in this study in MCF7 cells to complement the MTT results. It was found that dinaciclib followed by HAA_2020_ were the most clonogenic compounds compared to tamoxifen, and expectedly the combination of HAA_2020_ and dinaciclib showed the strongest clonogenic activity, followed by the combination of the three compounds. Moreover, HAA_2020_ was previously reported to exert potential Hsp90 inhibition activity [23], thus this was taken as another determinant for the selection of the best combination against MCF7 cells, because of the central role of Hsp90 in cell cycle progression. HAA_2020_ and dinaciclib were further taken for cell cycle and apoptosis mechanistic investigations. Each of the two compounds and their combination showed G_1_ cell cycle arrest. In addition, HAA_2020_ and dinaciclib together inhibited CDK2, cyclin D1, and PCNA, all leading to MCF7 cell cycle arrest, using Western blotting and immunofluorescent imaging assays. The combination of HAA_2020_ and dinaciclib also exhibited pronounced apoptosis in MCF7 cells, using the annexin V FITC/PI assay. In addition, it caused inhibition of VEGFR-2 and Hsp90.

Based on the results of the PA analysis using PamChip peptide microarray-based kinase activity profiling, it can be concluded that the Hsp90-mediated VEGFR-2 pathway is an important target, beside the importance of the cell cycle G_1_/S phase and its related growth regulators CDK2/cyclin D1 in addition to the importance of the phosphorylation processes in the tumorigenesis of Saudi luminal-A breast cancer samples. HAA_2020_ and dinaciclib were selected for combination studies along with tamoxifen, which revealed potent synergistic cytotoxic and clonogenic activities in MCF7 cells. The combination also showed G_1_ cell cycle arrest activity mediated with CDK2, cyclin D1, and PCNA inhibition. For the purpose of activity confirmation, the Hsp90/VEGFR-2 pathway was also shown by RT-PCR to be inhibited by the combination. This study highlighted one of the benefits of employing kinase activity profiling in drug discovery, and the study could be supporting evidence for the synergy between multi-TKIs and CDK inhibitors, which may help in the realization of new and effective second-line treatments for luminal-A breast cancer. Further in vivo and efficacy investigations are needed to provide more comprehensive preclinical information about the combination of HAA_2020_ and dinaciclib.

## 4. Materials and Methods

### 4.1. Collection, Preparation of Clinical Samples, and Ethical Clearance

BC biopsies (~2 × ~30 μm) were randomly collected from Saudi women diagnosed with luminal-A breast cancer (*n* = 12), who attended the King Fisal Specialist Hospital and Research Center (KFSHRC), Riyadh-Saudi Arabia during April 2016-April 2017, prior to receiving treatment. Patients signed ethical clearance No (2140006). The characteristics of the patients and their samples are summarized in Table 1. Following collection, samples were immediately frozen and stored at −80 °C without the addition of RNALater to keep the kinases intact.

### 4.2. Compounds and Reagents

**Figure 10:**
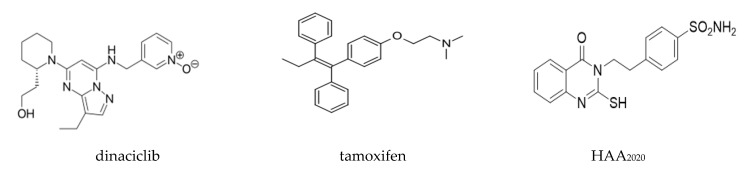
Molecular structures of dinaciclib, tamoxifen, and HAA_2020._

Dinaciclib and tamoxifen were purchased from Selleckchem, Houston, TX, USA. HAA_2020_ was thankfully provided by our collaborating group [22] (Figure 10). All reagents and kits used in this study were purchased from Sigma-Aldrich (St. Louis, MO, USA) unless another manufacturer is shown.

### 4.3. Cell Culture

The MCF7 cell line (breast adenocarcinoma, ER^+^/PR^+^/Her2^-^/EGFR^+^/VEGF^+^ (A, B, C, D)/VEGFR^+^ (1, 2, 3), cyclin D1, Hsp90, CK18 [46,47,48,49,50]; and MRC5 (normal non-transformed fibroblast) were obtained from the ATCC. Two types of media (RPMI-1640 and EMEM, both supplemented with 10% FBS, 1% Antibiotic-Antimycotic: all from Gibco) were used for sub-culture of MCF7 and MRC5 cells, respectively. Cells were kept at 100% humidity, 37 °C, and 5% CO_2_ for up to 10 passages. Mycoplasma was tested every other month using the bio-luminescence kit (Myco Alert sample detection kit; Lonza, Switzerland), and was read by a multi-plate reader (Synergy HT, BioTek, Winooski, VT, USA).

### 4.4. PamChip Peptide Microarray-Based Kinase Activity Profiling Arrays

Each fresh frozen breast tissue sample was lysed (lysis buffer: M-PER Mammalian protein extraction reagent # 78503; Halt phosphatase inhibitor cocktail # 78420; and protease inhibitor cocktail # 78415, 100 μL: 99.0: 0.5: 0.5, respectively; all from Thermo Fisher Scientific, Waltham, MA, USA), and incubated for 30 min at 0 °C. Samples were then centrifuged at 16,000 xg for 15 min at 0 °C. The lysates were stored in aliquots at −80 °C. Then, the protein concentration was determined with the Bradford assay (Thermo Fisher Scientific, Waltham, MA, USA). PTK activity was determined essentially as described by Chirumamilla et al., and STK activity as described by Hilhorst et al. [20,21]. Signal quantification of the peptides on the microarrays was done in Bionaviagor 6.3 (PamGene International BV, ‘s-Hertogenbosch, The Netherlands). Peptides on the PamChips are named after the protein that contains this sequence. However, the peptide sequence can be present in multiple proteins. To identify proteins that contain (part of) the peptide sequences on the chips, the peptides were blasted against UniProt. When the sequence homology was > 0.8, the protein was included into the data set that was used as input for Metacore analysis.

#### Data Interpretation

Data Interpretation was performed in Metacore (Clarivate Analytics, Cortellis, Philadelphia, PA, USA). UniProt IDs associated with peptides from both the PTK and STK arrays were uploaded into Metacore (Supplementary Material file S3). Analysis of the canonical pathways, networks, and GO processes was performed with selected proteins based on the signal intensities. For pathway analysis, UniProt IDs were projected on maps of canonical pathways that were created based on published peer-reviewed literature. The chance of finding a certain number of UniProt IDs on a canonical pathway was calculated and expressed as a *p*-value. The pathways were ranked by -log (*p*-Value: > 4 considered significant). Experimental data are visualized on the maps as blue (for downregulation) and red (upregulation) histograms (thermometers). The height of the histogram corresponds to the relative value for a particular protein.

### 4.5. Cytotoxicity and Combination Studies

The cytotoxicity of HAA_2020_, tamoxifen, dinaciclib, and their combinations was investigated in this study. The MTT (3-(4,5-dimethylthiazol-2-yl)-2,5-diphenyltetrazolium bromide) assay was used as previously reported [51]. MCF7 or MRC5 cells were cultured in 96 well (3 × 10^3^/well), which were treated with 10 concentrations of the three compounds: HAA_2020_: 0–5000 nM, tamoxifen: 0–5000 nM, dinaciclib: 0–100 nM (DMSO maximum 0.1%; *n* = 3). Plates were incubated for 72 h, followed by addition of MTT for 3 h (Life technologies, Waltham, MA, USA). Then, DMSO (100 µL) was added to each media-free well. The absorbance was read on a multi-plate reader (BIORAD, PR 4100, Hercules, CA, USA). The IC_50_ values were determined using GraphPad Prism (San Diego, CA, USA), and they were used to determine the combination ratio to be used between two of the compounds or all of them together (each in five concentrations) according to Table 3. CompuSyn software (ComboSyn, Inc., Paramus, NJ, USA) was used for the determination of the combination index (CI) in MCF7 cells. The selectivity index (SI) for a given drug or combination was calculated by dividing its IC_50_ against MRC-5 cells/IC_50_ against MCF7 cells.

### 4.6. Clonogenic Survival Assay

The clonogenic survival is a viability assay, in which the ability of individual cancer cells to form progeny colonies, was assessed based on a previous report [52]. Briefly, MCF7 cells were seeded in 2 mL of media at low density (2 × 10^2^) in 6-well plates as duplicates. Plates were incubated at 37 °C overnight to allow attachment. Cells were treated with either HAA_2020_ 500 nM, tamoxifen 750 nM, and dinaciclib 5 nM, the combination of two of them or three of them together. Plates were incubated at 37 °C for 24 h. Medium and compound(s) suspension were then aspirated, and 2 mL of fresh medium were added. Plates were checked under the microscope every 2 days. After 14 days, cells of at least 50 were considered a colony. Medium was aspirated, and cells were washed with cold Phosphate buffered Saline (PBS), then cells were fixed with cold methanol for 5 min at room temperature. Cells were then stained with 0.5% *v*/*v* methylene blue in methanol: H_2_O (1:1) for 15 min. Colonies were washed with PBS and H_2_O. Plates were left to dry, before the final counting of colonies.

### 4.7. Cell Cycle Analysis

MCF7 cells (5 × 10^5^/well in a 6-well plate) were treated with either HAA_2020_ (500, 2500, 5000 nM), dinaciclib (5, 25, 50 nM), or their combination for 24 h. Cells were then fixed in 70% ethanol and processed for cell cycle analysis, after staining with propidium iodide (PI, Santa Cruz), as previously reported [53]. A BC-500, Beckman Coulter (Indianapolis, IN, USA), USA flow cytometer was used for analyzing a total of 20,000 cells, with the aid of Expo 32 software.

### 4.8. Determination of Apoptosis

Evading apoptosis is one of the most important hallmarks of cancer [54]. We quantified the apoptotic activity of HAA_2020_, dinaciclib, or their combination using annexin V FITC/PI, as previously mentioned [55]. MCF7 cells were seeded in 6-well plates at 5 × 10^5^ cells/well. Then, cells were treated with the following: HAA_2020_ (500, 2500, 5000 nM), dinaciclib (5, 25, 50 nM), and their combination. Following 24 h, cells and supernatant were collected, spun, and binding buffer was added to the pellets. Annexin V FITC (Invitrogen) was then added to each sample and incubated at room temperature in the dark for 20 min, followed by the addition of PI. Samples were then analyzed by flow cytometry (BC-500, USA).

### 4.9. Western Blotting

Identification of the level change of cell cycle proteins (CDK2 and Cyclin D1) was investigated in this study. The effect of HAA_2020_ (500, 2500, 5000 nM), dinaciclib (5, 25, 50 nM), and their combination in MCF7 cells was tested following a previous report [23]. Briefly, MCF7 cells (1 × 10^6^ cells/well of a 6-well plate) were treated for 24 h. The lysis buffer was used to isolate total proteins, while the Bradford method was used to determine their concentration, which were then electrophoresed using a polyacrylamide gel and transferred to membrane. The membranes were incubated with CDK2 (# 2561, 1:1000, Cell signaling) and Cyclin D1 antibodies (# 2922, 1:1000, Cell signaling) for 2 h at room temperature and secondary antibody GAPDH (# 8884, 1:1000, Cell signaling) for 1 h. Visualization of the immunoreactivity was assessed using horseradish peroxidase (HRP)-conjugated secondary antibodies.

### 4.10. Real-Time PCR

The VEGF/VEGFR-2 are important for the survival of MCF7 cells [56,57,58], but VEGFR-2 cannot be detected by Western blotting [59]. Thus, the real-time RT-PCR platform (Applied Biosystems 7500 Fast Real Time PCR System) was used to quantify the gene expression of Hsp90 and VEGFR-2 **(**Table 4) in MCF7 cells (1 × 10^6^ cells), following treatment with HAA_2020_ (500, 2500, 5000 nM), dinaciclib (5, 25, 50 nM), and their combination for 24 h, as previously reported [23]. Briefly, the RNA isolation mini kit and the NanoDrop 2000 spectrophotometer (both from Thermo Scientific, Waltham, MA, USA) were used to determine the purity and concentration of the isolated RNA. The agarose gel (1%) electrophoresis was used to determine the purity of the isolated RNA and its integrity. The RevertAid First Strand cDNA Synthesis Kit (Thermo Fisher Scientific, Waltham, MA, USA) was used to synthesize the complementary DNA (cDNA) from the isolated RNA (2 µg) based on the manufacturer’s instructions. The RT-PCR was performed in a 96-well plate by the RT-PCR platform. The RT-PCR mixture was composed of 10 ng of cDNA, 10 µL of 2X SYBR Green I Master mix, 0.4 µM of each human primer Hsp-90, VEGFR-2, and GAPDH as the housekeeping gene (Applied-Biosystems, Thermo Fisher Scientific, Waltham, MA, USA), in addition to PCR-grade water up to a total volume of 20 µL. The RT-PCR program involved 45 cycles of denaturation at 95 °C for 15 s followed by annealing/extension at 60 °C for 60 s. All RT-PCR reactions were performed in triplicates and repeated 3 times (included a negative control: no template). To evaluate the genes’ expression, we used the standard comparative methods (ΔCt).

### 4.11. Immunofluorescence Staining

The inhibition of cyclin D1 and PCNA was confirmed by immunofluorescence staining. MCF7 cells (5 × 10^3^/chamber) were treated with HAA_2020_ (500 nM), dinaciclib (5 nM), and their combination for 24 h, according to a previous report [60]. EVOS FL microscopy (Thermo Fisher Scientific, Waltham, MA, USA) was used for slide examination. Digital images were taken with a 40× objective.

### 4.12. Statistics

Statistical differences were assessed by one-way Anova with the Tukey’s post-hoc multiple comparison test. *p* < 0.05 (*), *p* < 0.01 (**), *p* < 0.001 (***), and *p* < 0.0001 (****) were taken as significant.

## Figures and Tables

**Table 1:** Characteristics of the Saudi BC patients and their clinical samples.

**Age**	***n***	**Tumor size (cm)**	***n***
40–49	6	0–1.9	-
50–59	2	2–3.9	9
60–69	3	4–5.9	2
70–79	1	6–7.9	1
**gender**	***n***	**Position**	***n***
female	12	right	6
male	0	left	6
**Grade**	***n***	**Histology**	***n***
II	8	ductal	10
III	4	lobular	2
**ER%**	***n***	**PR%**	***n***
1–19	-	1–19	7
20–39	1	20–39	1
40–59	-	40–59	-
60–79	6	60–79	1
80–99	5	80–99	3
**Ki-67%**	***n***		
1–19	9		
20–39	1		
40–59	2		
60–79	-		
80–99	-		

**Table 2:** Top three networks found in the 12 luminal-A Saudi samples.

No	Network Name	Processes	Size	Target	Pathways	*p*-Value	zScore	gScore
1	VEGFR-2, cRaf-1, FAK1, c-Src, VEGFR-3	Transmembrane receptor protein tyrosine kinase signalling pathway (77.6%), enzyme linked receptor protein signalling pathway (81.6%), locomotion (85.7%), cell morphogenesis (73.5%), cell projection morphogenesis (67.3%).	52	16	56	4.65 × 10^−36^	70.29	140.29
2	TrkA, LAT, Syk, c-Src, Fer	Peptidyl-tyrosine phosphorylation (58.1%), peptidyl-tyrosine modification (58.1%), transmembrane receptor protein tyrosine kinase signalling pathway (65.1%), positive regulation of ERK1 and ERK2 cascade (55.8%), regulation of ERk1 and ERK2 cascade (58.1%).	53	29	3	1.25 × 10^−74^	128.86	132.61
3	Ephrin-A receptor 3, YES, Ephrin-A receptor 2, Fer, p130	Transmembrane receptor protein tyrosine kinase signalling pathway (50.0%), regulation of phosphorylation (68.8%), chemotaxis (47.9%), taxis (47.9%), regulation of protein phosphorylation (64.6%).	50	20	5	2.28 × 10^−47^	88.80	95.05

**Table 3:** IC_50_ values (72 h, nM), combination index, and selectivity index of HAA_2020_, tamoxifen, dinaciclib, and their combinations.

Drug(s) nM				MCF7		MRC5
HAA_2020_	Tamoxifen	Dinaciclib	IC_50_	*CI	IC_50_	**SI
125			480 ± 30		15221 ± 169	32
250						
500				-		
2500						
5000						
	187.5		738 ± 54		18450 ± 213	25
	375					
	750			-		
	3750					
	7500					
		1.25	4 ± 0		250 ± 14	62
		2.5				
		5		-		
		25				
		50				
HAA_2020:_ Tam (1:1.5)			207 ± 50		4558 ± 150	22
500	750			0.964		
2500	3750			0.823		
5000	7500			0.811		
HAA_2020:_ Din (100:1)			1 ± 0		105 ± 11	105
500		5		0.463		
2500		25		0.301		
5000		50		0.260		
Tam: Dina (150:1)			830 ± 91		16600 ± 390	20
	750	5		>1		
	3750	25		0.954		
	7500	50		0.901		
HAA_2020:_ Tam: Dina (100:150:1)			3 ± 0		153 ± 29	51
500	750	5		0.810		
2500	3750	25		0.714		
5000	7500	50		0.680		

*CI: Combination index at Fa = 0.9. **SI: selectivity index = IC_50_ value against normal MRC-5 cells/IC_50_ value against MCF7. IC_50_ values shown in mean ±SD (*n* = 3). Experiments were repeated three times.

**Table 4:** Sequence of Hsp90, VEGFR-2, and GAPDH primers.

Gene	Sequence
**Hsp90**	F: TTGGTTACTTCCCCGTGCTG R: GCCTTTTGCCGTAGGGTTTC
**VEGFR-2**	F: TGATACTGGAGCCTACAAGTGCTT R: CCTGTAATCTTGAACGTAGACATAAATGA
**GAPDH**	F: AGGTCGGTGTGAACGGATTTG R: TGTAGACCATGTAGTTGAGGTCA

## Supplementary Materials

The following are available online, Figure S1: Key of pathway analysis symbols, Table S2: Enrichment analysis by GO processes, Excel file S3: Breast phosphorylation by tyrosine kinase (PTK) and serine/threonine kinase (STK) data, Figure S4: Histograms of cell cycle analysis in MCF7 cells, Figure S5: Histograms showing detection of apoptosis in MCF7 cells (24 h).

## Abbreviations

17-AAD	17-*N*-allylamino-17-demethoxygeldanamycin
AIs	Aromatase inhibitors
AIB1	Amplified in breast cancer 1
ATM	Ataxia Telangiectasia Mutated
BC	Breast cancer
CDK	Cyclin dependent kinases
CI	Combination index
CREB5	CAMP Responsive Element Binding Protein 5
CREB3L4	CAMP Responsive Element Binding Protein 3 Like 4
DMSO	Dimethyl Sulfoxide
EFGR	Epidermal growth factor receptor
ErbB2	Erb-B2 Receptor Tyrosine Kinase 2
ER	Estrogen receptor
ESR1	Estrogen receptor 1
FAK1	Focal adhesion kinase 1
FITC	Fluorescein isothiocyanate
FOXM1	Forkhead box protein M1
GAPDH	Glyceraldehyde 3-phosphate dehydrogenase
HAA_2020_	compound 1 (4-(2-(4-Oxo-2-thioxo-1,4-dihydroquinazolin-3(2*H*)-yl)ethyl) benzene sulfonamide)
Her2	Human epidermal growth factor receptor 2
Hsp90	Heat shock protein 90
Hsp90i	Heat shock protein 90 inhibitors
HR	Hormone receptor
IGFBP2	Insulin-like growth factor (IGF) binding protein 2
MAPK	Mitogen activated protein kinase
MRAS	Muscle RAS oncogene
PA	Pathway analysis
PCNA	Proliferating cell nuclear antigen
PI	Propidium iodide
PI3K/AKT	Phosphoinositide-3-kinases/Protein kinase B
PIK3C2G	Phosphatidylinositol-4-Phosphate 3 Kinase Catalytic Subunit Type 2 Gamma
PKC	Protein kinase C
PLC gamma 1	Phospholipase C, gamma 1
RhoA	Ras homolog family member A
RoCK-1	Rho Associated Coiled-Coil Containing Protein Kinase 1
PR	Progestrone receptor
RT-PCR	Reverse transcription polymerase chain reaction
SERDs	Selective estrogen receptor degraders
SERMs	Selective estrogen receptor modulators
SI	Selectivity index
SRC	Proto-oncogene tyrosine-protein kinase
TKI	Tyrosine kinase inhibitors
TRAM-1	Translocation Associated Membrane Protein 1
VEGF	Vascular endothelial growth factor
VEGFR	Vascular endothelial growth factor receptor
VEGFR-2	Vascular endothelial growth factor receptor-2
