# Supplementary material for: Significance of Targeting VEGFR-2 and Cyclin D1 in Luminal-A Breast Cancer
Source: Molecules. 2020 Oct 10;25(20):4606. doi: 10.3390/molecules25204606 (PMC7594023; doi:10.3390/molecules25204606)
Supplement: Supplementary file 1 [file molecules-25-04606-s001.zip › molecules-943282-SM-CONVERSION/Supplementary material S1-S5-conversion.docx]

**Supplementary Materials**

**Figure S1.** Key of pathway analysis symbols.

**Table S2.** Enrichment analysis by GO processes

| **Processes** | **Network Objects from Active Data** | **Total** | ***p*-Value** |
| --- | --- | --- | --- |
| protein phosphorylation | PDGF-R-beta, PDGF receptor, ALS2CR7, PDK (PDPK1), Ephrin-A receptor 3, Ephrin-A receptors, GCK(MAP4K2), Ephrin-B receptors, Ephrin-B receptor 2, p38gamma (MAPK12), p38 MAPK, UFO, Lyn, SFK, Ephrin-A receptor 8, Fyn, VEGFR-1, JNK(MAPK8-10), JNK1(MAPK8), MAPK8/9, PFTAIRE-1, DDR1, PDGF-R-alpha, p38beta (MAPK11), MYO3B, Lck, Ephrin-A receptor 6, FAK1, ENO, ErbB2, LAT, **CDK3**, Insulin receptor, YES, PCTK2, ZAP70, CaMK II, CaMK II beta, ROS1, PCTK1, **CDK2**, CaMK II gamma, JNK3(MAPK10), Wee1B, JAK1, Ephrin-A receptor 5, Paxillin, DDR2, Syk, JNK2(MAPK9), JAK2, c-Src, STK36, PI3K reg class IA, PI3K reg class IA (p85-alpha), PI3K reg class IA (p85), FRK, ITK, MER, FGFR2, CaMK II alpha, **CDK1** (p34), BCKD-kinase, c-Fes, CALM3, CALM2, Calmodulin, ERK5 (MAPK7), STK4, Ephrin-A receptor 7, Tyro3, Ephrin-B receptor 3, c-Cbl, TrkC, ROR1, ULK3, unc-51-like kinase 3 (C. elegans), FGFR1, IRR, VEGFR-2, p38delta (MAPK13), Ephrin-A receptor 1, Ephrin-B receptor 1, MYO3A, Myosin IIIA, Myosin IIIA, GLK(MAP4K3), DCAMKL1, Ephrin-A receptor 2, PCTK3, LTK, Pyk2(FAK2), STK3, IGF-1 receptor, EGFR, ErbB4, p120GAP, RET, MSP receptor (RON), **CDK5**, TrkA, FGFR3, Myelin basic protein, Hck, HGF receptor (Met), TEC, ENO1, VEGFR-3, Fer, CaMK II delta, ALK, Ephrin-B receptor 4, TrkB, c-Raf-1, Ephrin-A receptor 4, STAT4 | 1291 | 1.313 × 10^−91^ |
| phosphorylation | PDGF-R-beta, PDGF receptor, ALS2CR7, PDK (PDPK1), Ephrin-A receptor 3, Ephrin-A receptors, BCKD, GCK(MAP4K2), Ephrin-B receptors, Ephrin-B receptor 2, p38gamma (MAPK12), p38 MAPK, UFO, Lyn, SFK, Ephrin-A receptor 8, Fyn, VEGFR-1, JNK(MAPK8-10), JNK1(MAPK8), MAPK8/9, PFTAIRE-1, DDR1, PDGF-R-alpha, p38beta (MAPK11), MYO3B, Lck, Ephrin-A receptor 6, FAK1, ENO3, ENO, ErbB2, LAT, **CDK3**, Insulin receptor, YES, PCTK2, ZAP70, CaMK II, CaMK II beta, ROS1, PCTK1, **CDK2**, CaMK II gamma, JNK3(MAPK10), Wee1B, JAK1, Ephrin-A receptor 5, Paxillin, DDR2, Syk, JNK2(MAPK9), JAK2, c-Src, STK36, PI3K reg class IA, PI3K reg class IA (p85-alpha), PI3K reg class IA (p85), FRK, ITK, MER, FGFR2, CaMK II alpha, **CDK1** (p34), BCKD-kinase, c-Fes, CALM3, CALM2, Calmodulin, ERK5 (MAPK7), ESR1 (nuclear), ESR1 (mitochondrial), ESR, ESR1 (membrane), STK4, Ephrin-A receptor 7, Tyro3, Ephrin-B receptor 3, c-Cbl, TrkC, ROR1, ULK3, unc-51-like kinase 3 (C. elegans), FGFR1, IRR, VEGFR-2, p38delta (MAPK13), Ephrin-A receptor 1, Ephrin-B receptor 1, MYO3A, Myosin IIIA, Myosin IIIA, GLK(MAP4K3), DCAMKL1, Ephrin-A receptor 2, PCTK3, LTK, Pyk2(FAK2), STK3, IGF-1 receptor, EGFR, ENO2, ErbB4, p120GAP, RET, MSP receptor (RON), **CDK5**, TrkA, FGFR3, Myelin basic protein, Hck, HGF receptor (Met), TEC, ENO1, VEGFR-3, Fer, CaMK II delta, ALK, Ephrin-B receptor 4, TrkB, c-Raf-1, Ephrin-A receptor 4, STAT4 | 1691 | 3.775 × 10^−87^ |
| peptidyl-tyrosine phosphorylation | PDGF-R-beta, PDGF receptor, Ephrin-A receptor 3, Ephrin-A receptors, Ephrin-B receptors, Ephrin-B receptor 2, UFO, Lyn, SFK, Ephrin-A receptor 8, Fyn, VEGFR-1, DDR1, PDGF-R-alpha, Lck, Ephrin-A receptor 6, FAK1, ErbB2, Insulin receptor, YES, ZAP70, ROS1, Wee1B, JAK1, Ephrin-A receptor 5, Paxillin, DDR2, Syk, JAK2, c-Src, FRK, ITK, MER, FGFR2, c-Fes, Ephrin-A receptor 7, Tyro3, Ephrin-B receptor 3, TrkC, ROR1, FGFR1, IRR, VEGFR-2, Ephrin-A receptor 1, Ephrin-B receptor 1, Ephrin-A receptor 2, LTK, Pyk2(FAK2), IGF-1 receptor, EGFR, ErbB4, RET, MSP receptor (RON), TrkA, FGFR3, Hck, HGF receptor (Met), TEC, VEGFR-3, Fer, ALK, Ephrin-B receptor 4, TrkB, Ephrin-A receptor 4 | 226 | 1.345 × 10^−80^ |
| peptidyl-tyrosine modification | PDGF-R-beta, PDGF receptor, Ephrin-A receptor 3, Ephrin-A receptors, Ephrin-B receptors, Ephrin-B receptor 2, UFO, Lyn, SFK, Ephrin-A receptor 8, Fyn, VEGFR-1, DDR1, PDGF-R-alpha, Lck, Ephrin-A receptor 6, FAK1, ErbB2, Insulin receptor, YES, ZAP70, ROS1, Wee1B, JAK1, Ephrin-A receptor 5, Paxillin, DDR2, Syk, JAK2, c-Src, FRK, ITK, MER, FGFR2, c-Fes, Ephrin-A receptor 7, Tyro3, Ephrin-B receptor 3, TrkC, ROR1, FGFR1, IRR, VEGFR-2, Ephrin-A receptor 1, Ephrin-B receptor 1, Ephrin-A receptor 2, LTK, Pyk2(FAK2), IGF-1 receptor, EGFR, ErbB4, RET, MSP receptor (RON), TrkA, FGFR3, Hck, HGF receptor (Met), TEC, VEGFR-3, Fer, ALK, Ephrin-B receptor 4, TrkB, Ephrin-A receptor 4 | 229 | 3.549 × 10^−80^ |
| protein auto-phosphorylation | PDGF-R-beta, PDGF receptor, PDK (PDPK1), Ephrin-A receptors, Ephrin-B receptors, p38 MAPK, Lyn, SFK, Ephrin-A receptor 8, Fyn, VEGFR-1, DDR1, PDGF-R-alpha, MYO3B, Lck, FAK1, ErbB2, Insulin receptor, YES, ZAP70, CaMK II, CaMK II beta, ROS1, CaMK II gamma, JAK1, DDR2, Syk, JAK2, c-Src, FRK, ITK, FGFR2, CaMK II alpha, c-Fes, ERK5 (MAPK7), STK4, Tyro3, Ephrin-B receptor 3, TrkC, ULK3, unc-51-like kinase 3 (C. elegans), FGFR1, IRR, VEGFR-2, Ephrin-A receptor 1, Ephrin-B receptor 1, MYO3A, Myosin IIIA, Myosin IIIA, Pyk2(FAK2), IGF-1 receptor, EGFR, ErbB4, **CDK5**, TrkA, FGFR3, Hck, HGF receptor (Met), TEC, VEGFR-3, Fer, CaMK II delta, ALK, Ephrin-B receptor 4, TrkB, Ephrin-A receptor 4 | 262 | 2.063 × 10^−79^ |
| phosphate-containing compound metabolic process | PDGF-R-beta, PDGF receptor, ALS2CR7, PDK (PDPK1), Ephrin-A receptor 3, Ephrin-A receptors, BCKD, GCK(MAP4K2), Ephrin-B receptors, Ephrin-B receptor 2, p38gamma (MAPK12), p38 MAPK, UFO, Lyn, SFK, Ephrin-A receptor 8, Fyn, VEGFR-1, JNK(MAPK8-10), JNK1(MAPK8), MAPK8/9, PFTAIRE-1, DDR1, PDGF-R-alpha, p38beta (MAPK11), MYO3B, Lck, Ephrin-A receptor 6, FAK1, ENO3, ENO, ErbB2, LAT, **CDK3**, Insulin receptor, YES, PCTK2, p47-phox, NADPH oxidase, ZAP70, CaMK II, CaMK II beta, ROS1, PCTK1, **CDK2**, CaMK II gamma, JNK3(MAPK10), Wee1B, JAK1, Ephrin-A receptor 5, Paxillin, DDR2, Syk, JNK2(MAPK9), JAK2, c-Src, STK36, PI3K reg class IA, PI3K reg class IA (p85-alpha), PI3K reg class IA (p85), FRK, ITK, MER, FGFR2, CaMK II alpha, **CDK1** (p34), BCKD-kinase, c-Fes, CALM3, CALM2, Calmodulin, ERK5 (MAPK7), ESR1 (nuclear), ESR1 (mitochondrial), ESR, ESR1 (membrane), STK4, Ephrin-A receptor 7, Tyro3, Ephrin-B receptor 3, c-Cbl, TrkC, ROR1, ULK3, unc-51-like kinase 3 (C. elegans), FGFR1, IRR, VEGFR-2, p38delta (MAPK13), Ephrin-A receptor 1, Ephrin-B receptor 1, MYO3A, Myosin IIIA, Myosin IIIA, GLK(MAP4K3), PP2A cat (alpha), PP2A catalytic, DCAMKL1, Ephrin-A receptor 2, PCTK3, LTK, Pyk2(FAK2), STK3, IGF-1 receptor, EGFR, ENO2, ErbB4, p120GAP, RET, MSP receptor (RON), **CDK5**, TrkA, FGFR3, Myelin basic protein, Hck, HGF receptor (Met), TEC, PLC-gamma, PLC-gamma 1, ENO1, VEGFR-3, Fer, CaMK II delta, ALK, Ephrin-B receptor 4, TrkB, PP2A cat (beta), c-Raf-1, Ephrin-A receptor 4, STAT4 | 2757 | 9.505 × 10^−70^ |
| phosphorus metabolic process | PDGF-R-beta, PDGF receptor, ALS2CR7, PDK (PDPK1), Ephrin-A receptor 3, Ephrin-A receptors, BCKD, GCK(MAP4K2), Ephrin-B receptors, Ephrin-B receptor 2, p38gamma (MAPK12), p38 MAPK, UFO, Lyn, SFK, Ephrin-A receptor 8, Fyn, VEGFR-1, JNK(MAPK8-10), JNK1(MAPK8), MAPK8/9, PFTAIRE-1, DDR1, PDGF-R-alpha, p38beta (MAPK11), MYO3B, Lck, Ephrin-A receptor 6, FAK1, ENO3, ENO, ErbB2, LAT, **CDK3**, Insulin receptor, YES, PCTK2, p47-phox, NADPH oxidase, ZAP70, CaMK II, CaMK II beta, ROS1, PCTK1, **CDK2**, CaMK II gamma, JNK3(MAPK10), Wee1B, JAK1, Ephrin-A receptor 5, Paxillin, DDR2, Syk, JNK2(MAPK9), JAK2, c-Src, STK36, PI3K reg class IA, PI3K reg class IA (p85-alpha), PI3K reg class IA (p85), FRK, ITK, MER, FGFR2, CaMK II alpha, **CDK1** (p34), BCKD-kinase, c-Fes, CALM3, CALM2, Calmodulin, ERK5 (MAPK7), ESR1 (nuclear), ESR1 (mitochondrial), ESR, ESR1 (membrane), STK4, Ephrin-A receptor 7, Tyro3, Ephrin-B receptor 3, c-Cbl, TrkC, ROR1, ULK3, unc-51-like kinase 3 (C. elegans), FGFR1, IRR, VEGFR-2, p38delta (MAPK13), Ephrin-A receptor 1, Ephrin-B receptor 1, MYO3A, Myosin IIIA, Myosin IIIA, GLK(MAP4K3), PP2A cat (alpha), PP2A catalytic, DCAMKL1, Ephrin-A receptor 2, PCTK3, LTK, Pyk2(FAK2), STK3, IGF-1 receptor, EGFR, ENO2, ErbB4, p120GAP, RET, MSP receptor (RON), **CDK5**, TrkA, FGFR3, Myelin basic protein, Hck, HGF receptor (Met), TEC, PLC-gamma, PLC-gamma 1, ENO1, VEGFR-3, Fer, CaMK II delta, ALK, Ephrin-B receptor 4, TrkB, PP2A cat (beta), c-Raf-1, Ephrin-A receptor 4, STAT4 | 2884 | 2.410 × 10^−67^ |
| transmembrane receptor protein tyrosine kinase signaling pathway | PDGF-R-beta, PDGF receptor, PDK (PDPK1), Ephrin-A receptor 3, Ephrin-A receptors, Ephrin-B receptors, Ephrin-B receptor 2, p38gamma (MAPK12), p38 MAPK, UFO, Lyn, SFK, Ephrin-A receptor 8, Fyn, VEGFR-1, DDR1, PDGF-R-alpha, p38beta (MAPK11), Lck, Ephrin-A receptor 6, FAK1, ErbB2, LAT, Insulin receptor, YES, p47-phox, NADPH oxidase, ZAP70, ROS1, CD3, JAK1, Ephrin-A receptor 5, Paxillin, DDR2, Syk, JAK2, c-Src, PI3K reg class IA, PI3K reg class IA (p85-alpha), PI3K reg class IA (p85), FRK, ITK, FGFR2, c-Fes, Ephrin-A receptor 7, Ephrin-B receptor 3, c-Cbl, TrkC, ROR1, FGFR1, IRR, VEGFR-2, p38delta (MAPK13), Ephrin-A receptor 1, Ephrin-B receptor 1, Ephrin-A receptor 2, LTK, Pyk2(FAK2), IGF-1 receptor, EGFR, ErbB4, p120GAP, RET, MSP receptor (RON), TrkA, FGFR3, Hck, HGF receptor (Met), TEC, PLC-gamma, PLC-gamma 1, VEGFR-3, Fer, ALK, Ephrin-B receptor 4, TrkB, c-Raf-1, Ephrin-A receptor 4 | 695 | 1.263 × 10^−65^ |
| positive regulation of protein phosphorylation | PDGF-R-beta, PDGF receptor, PDK (PDPK1), Ephrin-A receptor 3, Ephrin-A receptors, GCK(MAP4K2), Ephrin-B receptors, Ephrin-B receptor 2, p38 MAPK, UFO, Lyn, SFK, Ephrin-A receptor 8, Fyn, VEGFR-1, JNK(MAPK8-10), MAPK8/9, DDR1, PDGF-R-alpha, p38beta (MAPK11), Lck, Ephrin-A receptor 6, FAK1, ErbB2, LAT, Insulin receptor, YES, p47-phox, NADPH oxidase, CaMK II, ROS1, JNK3(MAPK10), CD3, Ephrin-A receptor 5, Paxillin, DDR2, Syk, JNK2(MAPK9), JAK2, c-Src, PI3K reg class IA, PI3K reg class IA (p85-alpha), PI3K reg class IA (p85), Annexin II, MER, FGFR2, **CDK1** (p34), CALM3, CALM2, Calmodulin, ESR1 (nuclear), ESR1 (mitochondrial), ESR, ESR1 (membrane), STK4, Ephrin-A receptor 7, Tyro3, Ephrin-B receptor 3, TrkC, ROR1, FGFR1, IRR, VEGFR-2, Ephrin-A receptor 1, Ephrin-B receptor 1, GLK(MAP4K3), PP2A cat (alpha), PP2A catalytic, Ephrin-A receptor 2, LTK, Pyk2(FAK2), STK3, IGF-1 receptor, TCF7L2/ beta-catenin, Beta-catenin, PECAM1, EGFR, ErbB4, Epo receptor, p120GAP, RET, MSP receptor (RON), **CDK5**, TrkA, FGFR3, HGF receptor (Met), TEC, PLC-gamma, PLC-gamma 1, VEGFR-3, CaMK II delta, ALK, Ephrin-B receptor 4, TrkB, c-Raf-1, Ephrin-A receptor 4 | 1401 | 2.861 × 10^−62^ |
| cellular protein modification process | PDGF-R-beta, PDGF receptor, ALS2CR7, PDK (PDPK1), Ephrin-A receptor 3, Ephrin-A receptors, BCKD, GCK(MAP4K2), Ephrin-B receptors, Ephrin-B receptor 2, Annexin I, p38gamma (MAPK12), p38 MAPK, UFO, Lyn, SFK, Ephrin-A receptor 8, Fyn, VEGFR-1, JNK(MAPK8-10), JNK1(MAPK8), MAPK8/9, PFTAIRE-1, DDR1, PDGF-R-alpha, p38beta (MAPK11), MYO3B, Lck, HIST1H2BD, Histone H2B, HIST1H2BG, Histone H2, Ephrin-A receptor 6, FAK1, ENO, ErbB2, LAT, **CDK3**, Histone H2BO, Insulin receptor, YES, PCTK2, ZAP70, CaMK II, CaMK II beta, ROS1, HIST1H2BM, PCTK1, **CDK2**, H2BFQ, CaMK II gamma, JNK3(MAPK10), Wee1B, JAK1, Ephrin-A receptor 5, Paxillin, DDR2, Syk, HIST1H2BA, JNK2(MAPK9), HIST1H2BN, JAK2, c-Src, STK36, PI3K reg class IA, PI3K reg class IA (p85-alpha), PI3K reg class IA (p85), FRK, ITK, ZNF145, HIST1H2BL, MER, FGFR2, CaMK II alpha, **CDK1** (p34), BCKD-kinase, c-Fes, CALM3, CALM2, Calmodulin, ERK5 (MAPK7), ESR1 (nuclear), ESR1 (mitochondrial), ESR, ESR1 (membrane), STK4, Ephrin-A receptor 7, Tyro3, Ephrin-B receptor 3, c-Cbl, TrkC, ROR1, ULK3, unc-51-like kinase 3 (C. elegans), FGFR1, IRR, VEGFR-2, p38delta (MAPK13), Ephrin-A receptor 1, Ephrin-B receptor 1, MYO3A, Myosin IIIA, Myosin IIIA, GLK(MAP4K3), PP2A cat (alpha), PP2A catalytic, DCAMKL1, Ephrin-A receptor 2, PCTK3, LTK, Pyk2(FAK2), STK3, IGF-1 receptor, EGFR, ErbB4, p120GAP, RET, MSP receptor (RON), HIST1H2BK, Histone H2B type 1-K, Histone H2B type 1-K, **CDK**5, TrkA, FGFR3, Myelin basic protein, Hck, HGF receptor (Met), TEC, ENO1, VEGFR-3, Fer, HIST1H2BB, CaMK II delta, ALK, Ephrin-B receptor 4, TrkB, PP2A cat (beta), Desmoplakin, c-Raf-1, Ephrin-A receptor 4, STAT4, HIST1H2BJ | 4029 | 7.539 × 10^−62^ |

Link to the supplementary material **S3**= excel file

<https://1drv.ms/x/s!AuA2qju9wwwomVAXNqa51OxIj34U?e=vH03AE>

| 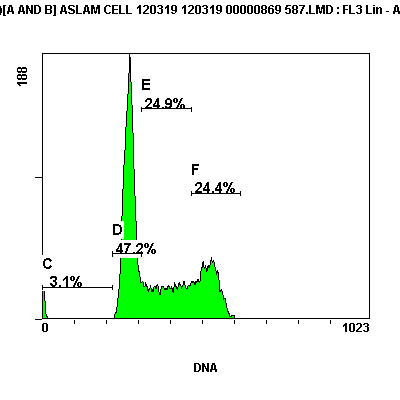 |  | |
| --- | --- | --- |
| control |  |  |
| 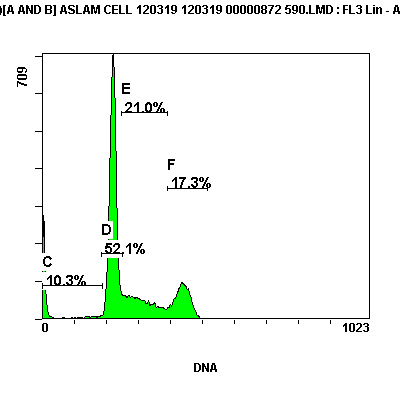 | 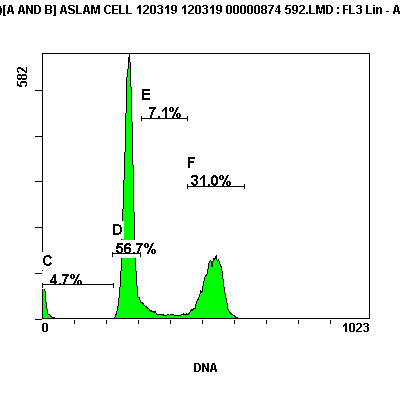 | 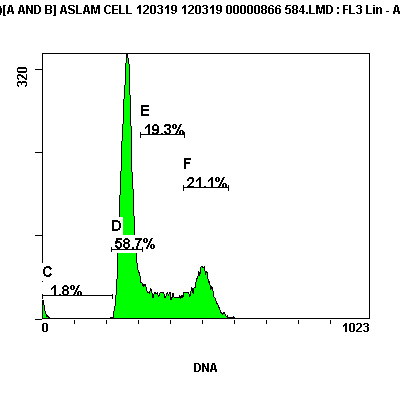 |
| HAA_2020_ 500 nM | HAA_2020_ 2500 nM | HAA_2020_ 5000 nM |
| 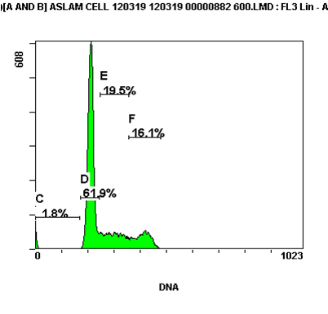 | 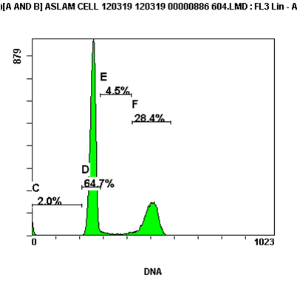 | 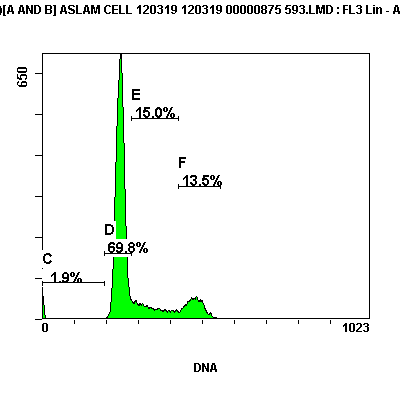 |
| dina 5 nM | dina 25 nM | dina 50 nM |
| 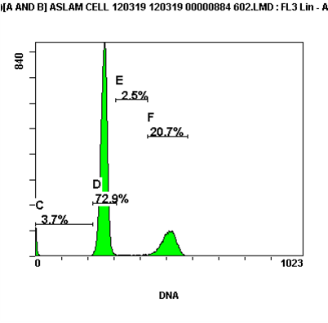 | 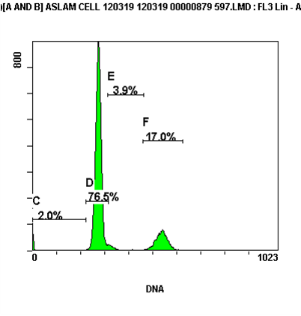 | 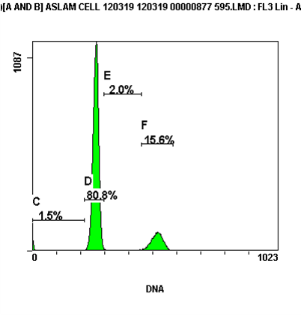 |
| HAA_2020_ 500 nM + dina 5 nM | HAA_2020_ 2500 nM + dina 25 nM | HAA_2020_ 5000 nM + dina 50 nM |

**Figure S4.** Histograms of cell cycle analysis in MCF7 cells. Cells were treated for 24 h with either HAA_2020_ (500, 2500, 5000 nM), dinaciclib (5, 25, 50 nM) or their combinations. Histograms of 20,000 events acquired on a BC-500 flow cytometer and analyzed by Expo 32 software, x-axis: DNA content, y-axis: % of cell number.

| 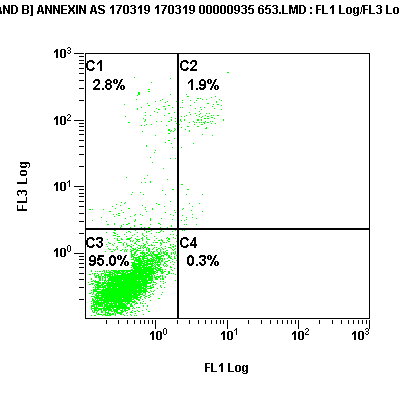 |  | |
| --- | --- | --- |
| control |  | |
| 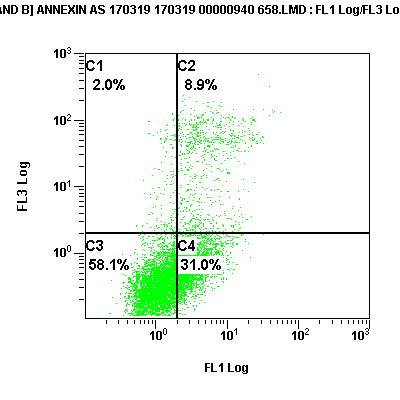 | 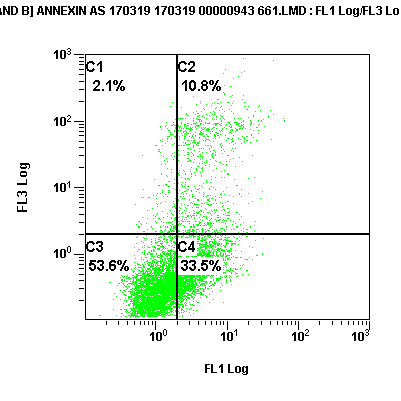 | 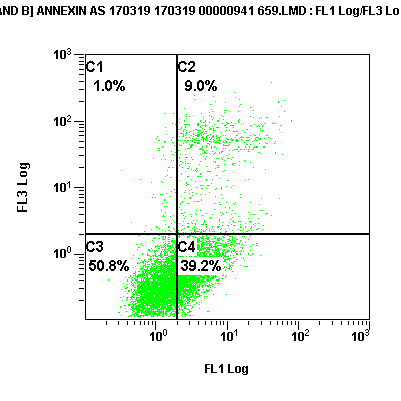 |
| HAA_2020_ 500 nM | HAA_2020_ 2500 nM | HAA_2020_ 5000 nM |
| 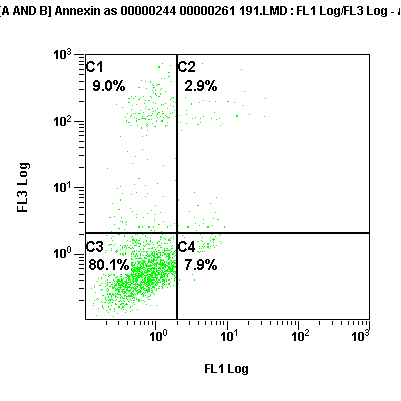 | 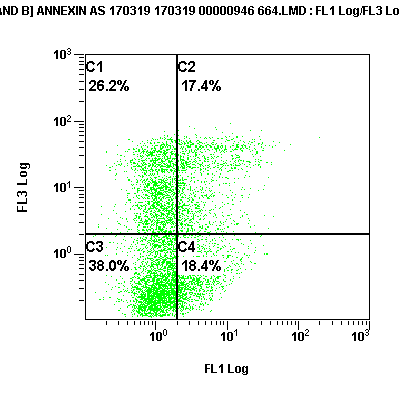 | 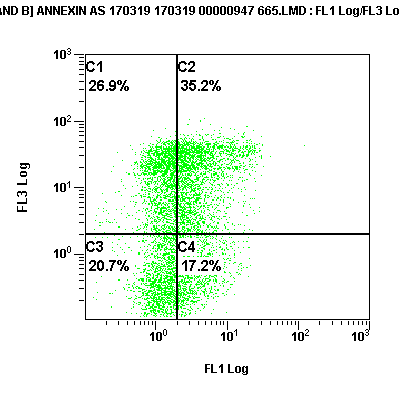 |
| dina 5 nM | dina 25 nM | dina 50 nM |
| 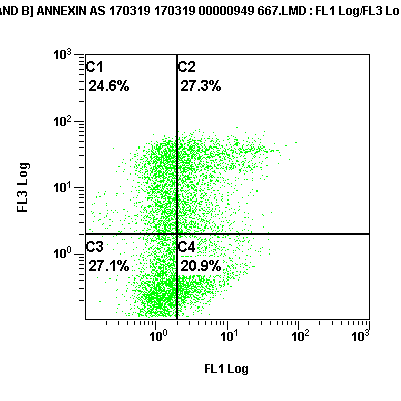 | 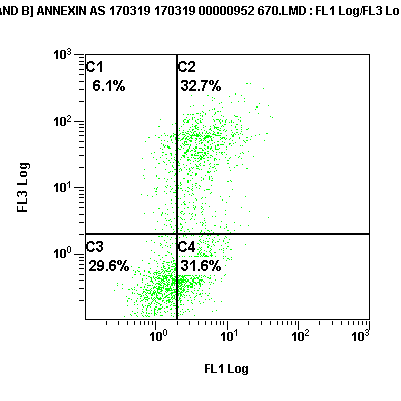 | 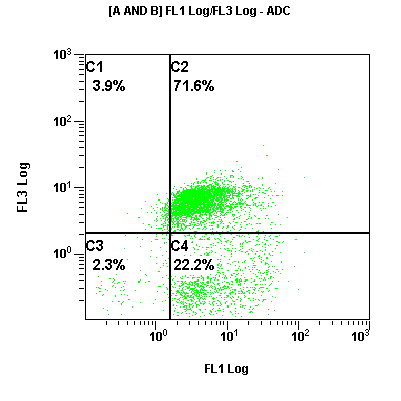 |
| HAA_2020_ 500 nM + dina 5 nM | HAA_2020_ 2500 nM + dina 25 nM | HAA_2020_ 5000 nM + dina 50 nM |

**Figure S5.**  Histograms showing detection of apoptosis in MCF7 cells (24 h). Cells were treated with either HAA_2020_ (500, 2500, 5000 nM), dinaciclib (5, 25, 50 nM) or their combinations. Cells were stained with annexin V FITC/PI. A total of 20,000 single-cell events were acquired on a BC-500 flow cytometer and analyzed by Expo 32 software. Cell staining status: necrotic cells (C1: annexin V−/PI+), late apoptotic cells (C2: annexin V+/PI+), live cells (C3: annexin V−/PI−), early apoptotic cells (C4: annexin V+/PI−). Annexin V (x-axis), and PI staining (y-axis).
